# 12 Tips for Centering Patient Voices through “Lived Experience Panels” in Preclinical Undergraduate Medical Education

**DOI:** 10.12688/mep.20260.1

**Published:** 2024-04-17

**Authors:** Premal Patel, Nadia Ahmed

**Affiliations:** 1 Department of Medicine, The University of Texas Medical Branch Health, Galveston, Texas, 77550, USA

**Keywords:** lived experience, narrative medicine, undergraduate medical education, storytelling

## Introduction

Authentically listening to the real-life experiences of patients occupies a unique space at the convergence of cultivating person-centered care, nurturing empathy, fostering rapport and connection and reflecting on what it means to be a physician for learners in preclinical undergraduate medical education. As a crucial branch point from which multiple curricula can be developed, intentionally integrating patient voices into formal medical education curricula early on offers benefits to both learners and patients.

Patients, acting as educators, self-experts and storytellers, contextualize their illness within the fabric of their lives. Leveraging the connection of storytelling, they find empowerment in sharing their life histories and the opportunity to sculpt their own narratives. Their status for learners rises from someone that is acted *upon* by health care professionals to someone who has *agency* and is a fully multidimensional person with hopes, fears, and nuance inherent in their humanity. Learners gain invaluable insights into the patient's experience of living with a specific disease or caring for someone with a chronic illness, understanding the profound impact of social determinants of health and psychosocial factors ^1– 3^.

Incorporating patient voices into medical education has many far-reaching benefits for learners. They gain insight into the patient experience, perspective of disease and health care, recognize the social context of illness, and acquire an understanding of the broader culture of health, healthcare, and medicine. Providing space for uninterrupted storytelling allows faculty to demonstrate role modeling of communication skills, empathy, and professional attitudes while reinforcing concepts of person-centered care ^4– 6^.

Patient narratives serve as a powerful tool not only for students, but also for the patients themselves. Those who are willing to share their stories often experience improved self-esteem, feelings of empowerment, and the ability to positively shape future patient experiences by educating healthcare professionals. By reflecting on their illness experience, patients can reframe and sculpt their illness narrative, gain deeper insight into their health, and seek support from peers. The prospect of shaping learner attitudes and beliefs can provide great personal satisfaction to the patient ^6– 8^.

At the University of Texas Medical Branch at Galveston, we created a series of lived experience panels (i.e LEPs) featuring real patients, sometimes alongside their caregivers and interprofessional care team, who shared their life stories with vulnerability, honesty, and bravery. We created different panel sessions on the themes of Incarceration, LGBTQI+ care, chronic disease, and disability that were facilitated by experts. Table 1 shows the session pre-work and format (see Table 1). LEP session themes can be expanded to include any relevant topics centered on the patient experience. The LEPs can serve as a model to integrate patient voices to stimulate further conversation and curricula on a wide range of topics and competencies.

**Table 1.** LEP sessions at UTMB.

Session Theme	Pre-work for students	Session Format
Incarceration	• Read Mass Incarceration Threatens Health Equity in America article	1. Small Group Discussion with Students over pre-prepared questions 2. Facilitated Discussion with Panelists moderated by faculty (4 panelists: 1 physician, 3 policy experts. All policy experts with experience in justice system themselves or secondary as family members) 3. Q&A from Students (moderated by faculty)
LGBTQI+ *	• Read a document on gender, sexuality, pronouns, etc.	1. Introduction of all participants 2. Moderated discussion by pre-selected prompts for all 3. Q&A with students
Chronic Disease	• View taking care of Complex Patient Powerpoint • Read Importance of Physician Listening article	1. Introduction of all participants 2. Mother tells the story of her daughter 3. Care team tells their perspective (MD, OT, SW) 4. Mom signs off 5. Q&A with students and care team
Disability	• View a video on www.positiveexposure.org and answer prompts	1. Short introduction 2. Introduction of all patients and their families 3. Each family tells their story 4. Families are invited to give advice to future doctors 5. Brief Q&A 6. Didactic of best practices

*This session was followed by a formal Allies in Health LGBTQI+ training

In the subsequent twelve tips, we aim to offer guidance for effectively implementing lived experience panels in medical school curricula.

## 12 Tips

 1. Curate panelists thoughtfully: Carefully select panelists representing diverse patient experiences and perspectives. Consider inviting people from outside of the medical community to broaden the scope of the conversation.
 2. Consider timing: When scheduling sessions, take into account the mental, cognitive and emotional bandwidth of the students in conjunction with their other coursework to ensure an optimal learning environment. Align the sessions with the curriculum and the students’ current learning objectives for maximal impact.
 3. Create conditions for vulnerability: Create an environment that allows for vulnerability from both the panelists and the students. Frame the session with thoughtful, pre-curated questions that encourage meaningful dialogue and open exchange.
 4. Promote patient expertise: Cultivate a shared decision-making framework placing patients at the center of their care that regards them as their own experts.
 5. Empower historically marginalized communities: Carefully curate panelists from marginalized communities and allow them the space to share their narratives authentically.
 6. Leverage virtual platforms: Utilize virtual platforms to create comfort for panelists, expand recruitment of panelists from larger geographic areas and offer students a glimpse of patients in their own environment. Additionally, ensure sessions are not recorded to allow for greater confidentiality.
 7. Facilitate the discussion: Develop prompts to guide the panelists and ensure active facilitation, redirecting biomedical questions to focus on the patient experience during question-and-answer part of the session.
 8. Prepare students beforehand: Assign preparatory activities such as readings, videos, and reflective writing prompts to ensure students are ready for the session. Additionally, create norms for engagement to ensure respectful engagement and civil discourse, preparing students for diverse perspectives and potential disagreement.
 9. Debrief with students: Implement mechanisms to check in with students emotionally impacted by the session and provide access to counseling if needed.
10. Prepare patient panelists beforehand: Ensure panelists understand the goal of the session, co-create the session, and have the opportunity to share their stories fully. Additionally, provide them with information on the level of training the students have received.
11. Debrief with patient panelists and curators: Allow for a debriefing period with panelists and curators to ensure the session goal was achieved, their experience was positive, and their unique perspectives were valued. Additionally, create a mechanism for students to show their appreciation.
12. Collaborate with your local community: Foster authentic relationships with people both inside and outside of the medical community and create a network in your local community to further enrich the lived experience panels.

## Conclusion

Integrating lived experience panels into medical education can have numerous benefits for both learners and patients. Thoughtful, intentional, and sensitive inclusion of these panels is essential for creating meaningful curricula. By incorporating patient voices into medical education, we can foster empathy, person-centered care, and authentic connection among learners, which has the potential to transform the physician-patient relationship.

## Data Availability

No data are associated with this article.
