# Supplementary material for: 12 Tips for Centering Patient Voices through “Lived Experience Panels” in Preclinical Undergraduate Medical Education
Source: MedEdPublish (2016). 2024 Apr 17;14:23. [Version 1] doi: 10.12688/mep.20260.1 (PMC11211732; doi:10.12688/mep.20260.1)
Supplement: Peer review history

# Reviewer report: Reviewer response for version 1

**DOI:** 10.21956/mep.21688.r37330

**Published:** 2024-06-27

**Authors:** Mantoa Mokhachane, Aviwe Mgobozi

**Affiliations:** 1 University of Witwatersrand, Johannesburg, South Africa; 2 Clinical Associates, School of Clinical Medicine, University of Witwatersrand, Johannesburg, Gauteng, South Africa

The article discusses an exciting and innovative pedagogical approach to health professions education. Strengthening the voices of patients through lived experience panels is beneficial to both patients and students. It is a useful method for patients as they gain deeper insights into their own experiences and illnesses. On the other hand, it is beneficial for students to practice patient centred consultations and obtain deeper insights into conditions, opinions and views from patients. This yields empathy and a greater connection.

 The 12 tips are useful to outline the considerations for implementing the lived experience panels for health professions education.

 It is unclear whether the 12 tips originate from a research study or whether it is based on the perceptions of the authors following the implementation of the lived experience panels.

 The paper aims to outline the “leveraging of the virtual platforms, skilful facilitation, student and patient preparedness, post-session support, and debriefing mechanisms”. However, the aim has been partially addressed as there are no practical measures suggested for the reader to fulfil the mentioned steps towards a successful lived experience panel. The aims and objectives were only mentioned in the abstract, it is not clear whether this was intentional because of word limit or whether the authors were avoiding repetition.

 It is insightful to see the various panels and sessions which were centred around various objectives and experiences namely incarceration, LGBTQI, Chronic disease, and disability. It is unclear how panellists were selected for each panel and the objectives the students were anticipated to obtain from each session.

 Sharing the logistics would be useful to the reader for replication of the method in different environments. The student numbers were not included and the administration requirements for the successful execution of the lived experience panel are unclear. In Table 1 LEP sessions; in the session format column, it is not clear whether patients are participants, especially the incarceration section. In the disability LEP session, it is not clear whether there is more than one family or not. If there is more than one family, it is not clear how their incorporation  is facilitated or handled.

 The sequence of the 12 tips would benefit the reader if there was a chronological order of implementing the lived experience panels, starting with considerations for the planning phase, the execution, and lastly the post-panel phase. This would enhance the flow and coherence upon reading the tips. In the third tip, it is mentioned that they ‘create conditions for vulnerability’, it is not clear how that is done. In the fourth tip patient’s expertise is meant to be promoted, as this was not explicit in the LEP sessions Table. How are the historically marginalized communities empowered, as mentioned in the fifth tip.

 The 12 tips are general measures and considerations. Linking the 12 tips to the lived experience panels as mentioned earlier in the article would increase the relatability of the article and ground the concept with practical measures based on the real activity. More examples are required so as to address the void of the reader’s curiosity on the practical steps taken to facilitate the activity, and larger pedagogical approach from the time of initiation to debriefing and assessing the impact of the event.

If evidence from practice is presented, are all the underlying source data available to ensure full reproducibility?

Partly

Is the topic of the practical tips discussed accurately in the context of the current literature

Yes

Are all factual statements correct and adequately supported by citations?

Yes

Are the conclusions drawn balanced and justified on the basis of the presented arguments?

Partly

Are arguments sufficiently supported by evidence from the published literature and/or the authors’ practice?

Yes

Reviewer Expertise:

Professionalism and Professional Identity formation. Medical Education

We confirm that we have read this submission and believe that we have an appropriate level of expertise to confirm that it is of an acceptable scientific standard, however we have significant reservations, as outlined above.

# Reviewer report: Reviewer response for version 1

**DOI:** 10.21956/mep.21688.r37328

**Published:** 2024-06-20

**Authors:** Matt Sibbald, Debbie Azim Fleming

**Affiliations:** 1 McMaster University, Hamilton, Ontario, Canada; 2 McMaster University, Hamilton, Ontario, Canada

This is a valuable addition to the literature. While including patient voices in medical education is well supported in the current literature, information on how to support and operationalize this process is limited. These tips add to the existing literature base. Consider the use of language (eg Groups that have been historically marginalized vs groups that have been marginalized): https://www.ama-assn.org/system/files/ama-aamc-equity-guide.pdf

*Are all factual statements correct and adequately supported by citations?*

  Citations provided in the article support including patient voices in medical education but do not specify or reference Lived Experience Panels (LEP) as a  model that can be an effective approach.  It would be helpful to know what led to introducing  LEP as a potential model for incorporating patient narratives.

*Are arguments sufficiently supported by evidence from the published literature and/or the authors’ practice?*

 Including evidence which supports the use of LEP and how the tips were developed would better support arguments.  For example, were students or panelists or faculty surveyed?  Tips offered could be strengthened by addressing: 

 · Ideal time periods for check-ins/debriefing with students and panelists 

 ·Consideration of compensation of marginalized speakers for their time 

 · Limits or challenges in using LEP

*If evidence from practice is presented, are all the underlying source data available to ensure full reproducibility?*

  Method or source data was not presented.  There was no indication if surveys or other methods which could be reproduced were used to gather evidence to develop the tips shared. 

 The relevance of Table 1 could be strengthened by the inclusion of objectives linked to medical education competencies.

*Are the conclusions drawn balanced and justified on the basis of the presented arguments?*

**Conclusions presented would be better supported by including in the body of the article the specific benefits of incorporating the LEP model in the curriculum.

If evidence from practice is presented, are all the underlying source data available to ensure full reproducibility?

Partly

Is the topic of the practical tips discussed accurately in the context of the current literature

Yes

Are all factual statements correct and adequately supported by citations?

Yes

Are the conclusions drawn balanced and justified on the basis of the presented arguments?

Yes

Are arguments sufficiently supported by evidence from the published literature and/or the authors’ practice?

Partly

Reviewer Expertise:

Undergraduate medical education; Clinical reasoning; Competency based education; Simulation based education

We confirm that we have read this submission and believe that we have an appropriate level of expertise to confirm that it is of an acceptable scientific standard, however we have significant reservations, as outlined above.

# Reviewer report: Reviewer response for version 1

**DOI:** 10.21956/mep.21688.r37043

**Published:** 2024-06-18

**Authors:** Briseida Mema

**Affiliations:** 1 Hospital for Sick Children, University of Toronto, Toronto, Canada

The authors present an established curriculum that involves patients' narratives in the preclinical setting with the goal of supporting holistic care. 

 The article is very well written, and the practical tips would be very helpful to educators who want to establish similar curricula. The topic is very important to the medical community and educators. 

 The only minor comment I have is at the last paragraph before the 12 Tips start, the word real patients should be replaced by patients (we know they are real).

 Thank you for the opportunity to read this paper.

If evidence from practice is presented, are all the underlying source data available to ensure full reproducibility?

Yes

Is the topic of the practical tips discussed accurately in the context of the current literature

Yes

Are all factual statements correct and adequately supported by citations?

Yes

Are the conclusions drawn balanced and justified on the basis of the presented arguments?

Yes

Are arguments sufficiently supported by evidence from the published literature and/or the authors’ practice?

Yes

Reviewer Expertise:

Medical humanities

I confirm that I have read this submission and believe that I have an appropriate level of expertise to confirm that it is of an acceptable scientific standard.
